# Delay discounting and under-valuing of recent information predict poorer adherence to social distancing measures during the COVID-19 pandemic

**DOI:** 10.1038/s41598-021-98772-5

**Published:** 2021-09-28

**Authors:** Alex Lloyd, Ryan McKay, Todd K. Hartman, Benjamin T. Vincent, Jamie Murphy, Jilly Gibson-Miller, Liat Levita, Kate Bennett, Orla McBride, Anton P. Martinez, Thomas V. A. Stocks, Frédérique Vallières, Philip Hyland, Thanos Karatzias, Sarah Butter, Mark Shevlin, Richard P. Bentall, Liam Mason

**Affiliations:** 1 Department of Psychology, Royal Holloway, University of London, Egham Hill, Egham, TW20 0EX England; 2 University of Manchester, Manchester, England; 3 University of Dundee, Dundee, Scotland; 4 University of Ulster, Ulster, Northern Ireland; 5 University of Sheffield, Sheffield, England; 6 University of Liverpool, Liverpool, England; 7 Trinity College Dublin, Dublin, Republic of Ireland; 8 National University of Ireland, Maynooth, Republic of Ireland; 9 Edinburgh Napier University, Edinburgh, Scotland; 10 University College London, London, England

**Keywords:** Psychology, Human behaviour

## Abstract

The COVID-19 pandemic has brought about unprecedented global changes in individual and collective behaviour. To reduce the spread of the virus, public health bodies have promoted social distancing measures while attempting to mitigate their mental health consequences. The current study aimed to identify cognitive predictors of social distancing adherence and mental health symptoms, using computational models derived from delay discounting (the preference for smaller, immediate rewards over larger, delayed rewards) and patch foraging (the ability to trade-off between exploiting a known resource and exploring an unknown one). In a representative sample of the UK population (*N* = 442), we find that steeper delay discounting predicted poorer adherence to social distancing measures and greater sensitivity to reward magnitude during delay discounting predicted higher levels of anxiety symptoms. Furthermore, under-valuing recently sampled information during foraging independently predicted greater violation of lockdown guidance. Our results suggest that those who show greater discounting of delayed rewards struggle to maintain social distancing. Further, those who adapt faster to new information are better equipped to change their behaviour in response to public health measures. These findings can inform interventions that seek to increase compliance with social distancing measures whilst minimising negative repercussions for mental health.

## Introduction

The COVID-19 pandemic has required radical behavioural changes worldwide, most notably the enacting of social distancing measures and nationwide lockdowns. While public adherence to these novel measures has generally been good^1^, their introduction in response to the first waves of the pandemic has been associated with elevated rates of mood and anxiety disorders^2^, and instances of non-compliance have hindered efforts to control the spread of the virus^3^. In light of new waves of infection, there is growing concern about the public’s ability to sustain these behaviours as the response to the pandemic becomes increasingly protracted. Public health bodies worldwide thus face a dual challenge: how to promote compliance with public health measures, while coping with the mental health consequences these measures may engender. Here we examine (a) whether differences in reward-based decision-making predict adherence to social distancing measures during the early acute and later established phases of the pandemic’s first wave; (b) which cognitive characteristics predict poorer mental health during the pandemic; and (c) how mental health symptoms impact the relationship between reward-based decision-making and social distancing adherence. Answers to these questions are crucial to inform public health policy and are in line with calls for the behavioural sciences to be at the heart of the national pandemic response to COVID-19^4,5^.

Adhering to social distancing guidelines during the pandemic requires individuals to forgo the temptation of immediate gratification (e.g., seeing friends, going to public places of leisure, etc.) in favour of obtaining significantly larger future rewards, both for themselves and society (e.g., reduction in the spread and impact of COVID-19, relaxation of restrictions on freedom of movement, etc.). The extent to which people place greater value on immediate rewards than on larger, but delayed rewards has been termed “delay discounting”^6^, which we measure in this study. Lower levels of delay discounting are typically associated with adaptive behaviours^7^ and have been associated with multiple health-promoting behaviours (including engaging in more frequent exercise and choosing to wear a seat belt while driving), lower rates of illness and greater longevity^8^. Conversely, higher levels of delay discounting predict a number of problematic health behaviours (including use of alcohol, tobacco and other drugs^9^), as well as poorer emotion regulation^10^ and higher levels of mood and anxiety symptoms^11^. In the context of the COVID-19 pandemic, participants’ intention to comply with social distancing policies declines as the length of time that these measures are mandated increases^12,13^, demonstrating delay discounting in this novel context. However, there is limited understanding about whether individual differences in delay discounting predicts adherence to this novel health behaviour.

Farsighted decisions can be influenced by the magnitude of the delayed reward^14,15^ with evidence that the rate of discounting declines if the magnitude of the delayed option is increased. The degree to which individuals are influenced by the magnitude of the delayed reward has also been shown to predict health behaviours^16^. For example, cigarette smokers modulate their delay discounting more as a function of reward magnitude compared to non-smokers^17^, and it has been demonstrated that an intervention to foster cognitive control (generating justifications for choices) can reduce sensitivity to the magnitude effect in delay discounting^18^.

With regards to mental health, our working hypothesis is that lockdown is associated with fewer opportunities for immediate rewards, and that steeper discounters may find it subjectively more aversive when immediate reward frequency is lower (compared to shallower discounters), leading to high levels of mood and anxiety symptoms. Therefore, delay discounting preferences may be informative in identifying individuals who struggle to adhere to social distancing guidelines and who are at greater risk of poor mental health outcomes as a result of lockdown. Stressful changes in the environment may in turn also impact delay discounting. Supporting this view, participants’ discounting became steeper in the aftermath of the Wenchuan earthquake, for example^19^.

While delay discounting measures how people prioritise long versus short-term outcomes for the future, there are separate cognitive characteristics that are responsible for how much priority individuals place on recent experiences. Rational models of decision-making contend that it is adaptive to weight recent reward outcomes strongly when in a dynamically changing environment^20^. In a volatile environment, previously learned associations between actions and outcomes become less certain^21^; consequently, the decision-maker should prioritise more recent information to guide their decisions because it is more likely reflects the current structure of the environment^20^. During the COVID-19 pandemic, where health-relevant information evolves rapidly^5^, optimal decision-making necessitates updating one’s beliefs quickly. To examine this, we leveraged a patch foraging task^12^ to measure individual differences in the weight placed on new information about the current structure of the environment (i.e. learning rate^22^). Patch foraging involves a trade-off between exploiting a known patch which gradually diminishes in resources versus exploring a novel patch with a fresh distribution of resources^12^. To maximise the intake of rewards, the decision-maker must learn the optimal point to leave the current patch to explore a novel one, which can be used to quantify the degree to which they weigh recent information to make decisions^23,24^.

However, the ability to adapt behaviour according to the structure of one’s environment has been shown to be disrupted by anxiety^7,26^. In the context of patch foraging, this would reflect a poor ability to adjust the point at which the forager leaves the current patch to changes in the quality of resources in the surrounding environment. A reduced ability to implement behavioural change is particularly problematic in the context of the COVID-19 crisis, which has required the population to adopt a series of novel behaviours^43^.

Based on the delay discounting and patch foraging literature, we pre-registered hypotheses that delay discounting would predict adherence to social distancing measures during the COVID-19 pandemic (*H1a,* two-tailed). Insofar as delay discounting is a stable characteristic^9,25^ that captures the degree to which individuals devalue delayed rewards, one might expect higher levels of delay discounting to predict poorer adherence to social distancing. However, given evidence that delay discounting increases in response to crises^10^, it could be that steeper discounting reflects an adaptive response to the changeable environment. As such, it is also possible that higher levels of discounting will predict *greater* adherence to social distancing. We additionally hypothesised that higher learning rates would predict greater social distancing adherence (*H1b*). Finally, we hypothesised that poorer mental health, including specific anxiety about the COVID-19 pandemic, would be predicted by higher levels of delay discounting (*H2a*) and a reduced ability to adapt the point at which they left patches between foraging environments (*H2b)*. To test the above, we utilised a scale measuring social distancing (Gibson-Miller et al., in prep; see Methods) and predicted that a dimensional representation of social distancing would be identifiable that would allow us to determine the degree to which participants were adhering to or violating the government rules around social distancing.

## Results

### Cognitive characteristics predicted adherence to social distancing during lockdown

Results of a factor analysis confirmed that a two-factor solution best fit the data regarding participants’ self-reported behaviours related to COVID-19 public health policies at the established phase of the pandemic. These factors captured two separate facets of behaviour: adherence to social distancing measures (e.g., stayed at least 2 m away from others in public) and active violation of lockdown guidance (e.g., gathered in a group of people). We subsequently conducted two regression analyses (using ordinary least squares, OLS) using social distancing adherence and active violation as the outcome variables. Parameters from the delay discounting task (baseline discount rate, c, and sensitivity towards reward magnitude, m), parameters from the patch foraging task (learning rate and ability to adjust foraging behaviour between the two environments), age, gender and income were entered as predictor variables in both regressions.

**Figure 1:**
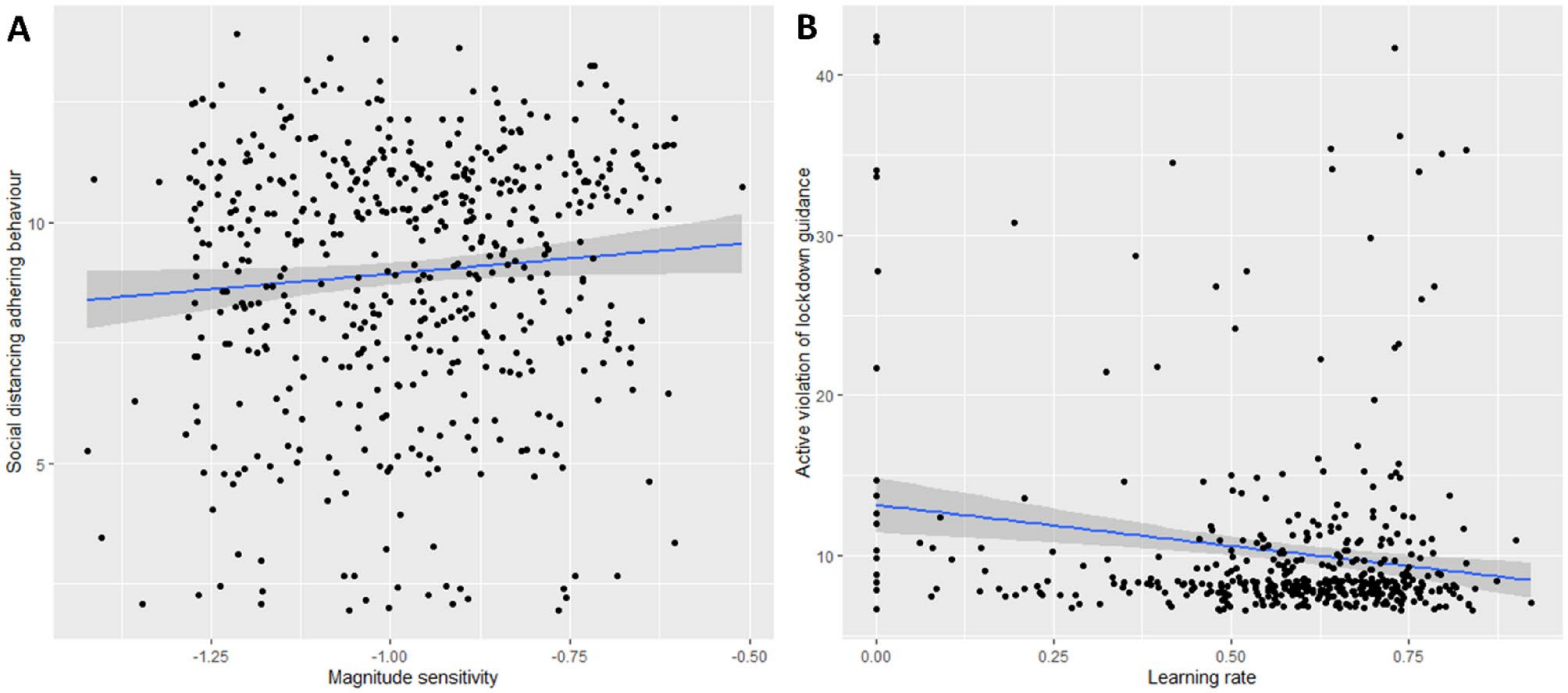
Scatterplots indicating the associations between cognitive and social distancing measures. (**A**) Participants who were more sensitive to reward magnitude (i.e., had lower values on the x-axis) reported lower levels of social distancing adherence (i.e., had lower values on the y-axis). (**B**) Participants who had a lower learning rate (i.e., had lower values on the x-axis) reported higher levels of active violation of lockdown policies (i.e., had higher values on the y-axis). Regression lines are highlighted in blue.

The overall model predicting social distancing adherence was significant *F*_(7,385)_ = 3.91, *p* < 0.001, R^2^ = 0.07. Lower levels of social distancing adherence were predicted by baseline discounting rate (β =  − 0.13, t =  − 2.48, *p* = 0.014) and sensitivity to reward magnitude (β = 0.16, t = 3.38, *p* = 0.002), indicating that steeper discounting and greater sensitivity to variations in reward magnitude predicted poorer adherence to social distancing guidance (see Fig. 1A). However, the learning rate (β =  − 0.02, t = 0.34, *p* = 0.734) and ability to adjust foraging behaviour between the two environments (β = 0.04, t = 0.83, *p* = 0.409) were not significant predictors. In addition, younger age (β = 0.13, t =  − 2.63, *p* = 0.009) and lower income (β = 0.11, t = 2.32, *p* = 0.032) predicted poorer social distancing adherence.

The regression model predicting active violation of lockdown guidance was also significant *F*_(7,385)_ = 2.85, *p* = 0.007, R^2^ = 0.05. Higher levels of active violation of lockdown guidance was predicted by lower learning rate (β =  − 0.11, t = 2.31, *p* = 0.024; see Fig. 1B) and younger age (β = 0.14, t = 4.12, *p* = 0.005), but not by the ability to adjust foraging behaviour (β =  − 0.05, t = 0.74, *p* = 0.323), sensitivity to reward magnitude (β =  − 0.08, t = 1.07, *p* = 0.099) or baseline discounting rate (β = 0.02, t = 0.98, *p* = 0.570).

### Delay discounting predicted mood and anxiety symptoms

**Figure 2:**
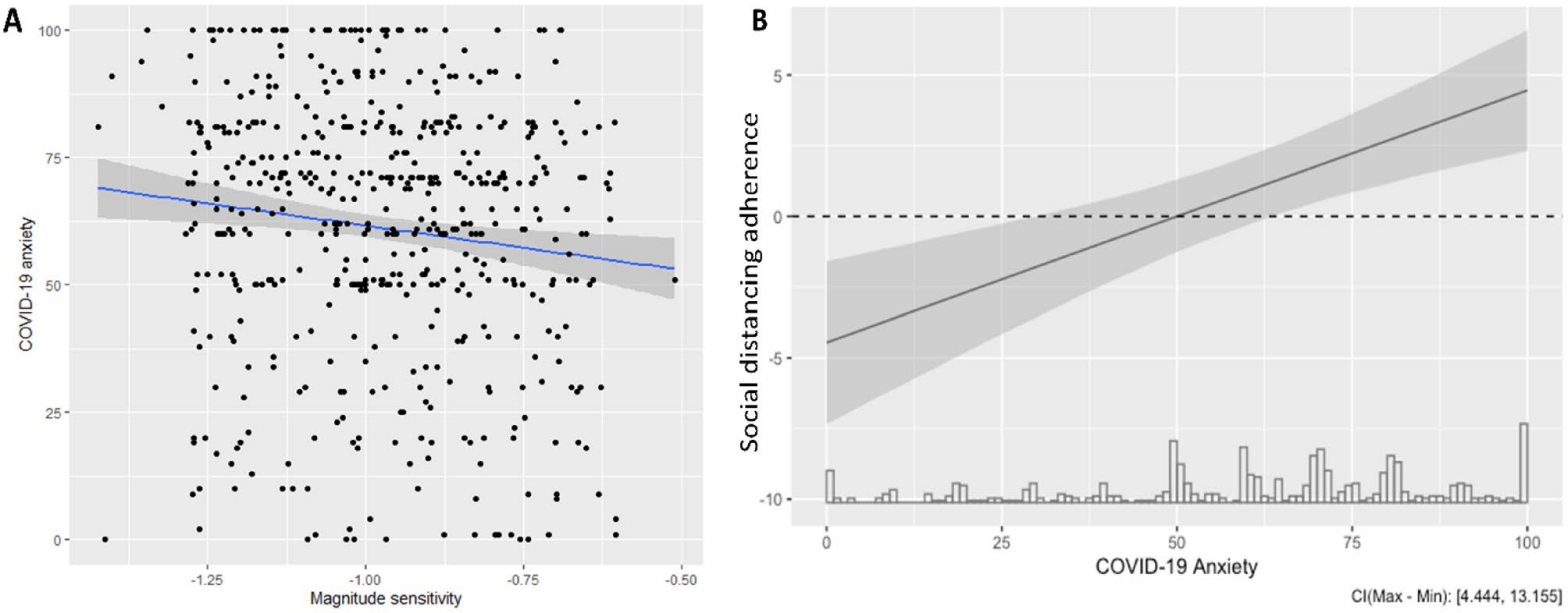
(**A**) Scatterplot indicating greater sensitivity to reward magnitude during delay discounting (i.e., lower values on the x-axis) predicted higher levels of specific COVID-19 anxiety. The regression line is highlighted in blue. (**B**) Plot demonstrating the conditional effect of sensitivity towards reward magnitude during delay discounting on social distancing adherence by levels of specific COVID-19 anxiety.

We conducted separate OLS regressions for COVID-19 specific anxiety, depression, and generalised anxiety. Delay discounting and patch foraging parameters were entered as predictors into each regression alongside age, gender, and income. The regression model predicting COVID-19 anxiety was significant *F*_(7,385)_ = 2.69, *p* = 0.01, R^2^ = 0.05. Greater sensitivity towards reward magnitude predicted higher levels of specific anxiety around COVID-19 (β =  − 0.17, t = 3.34, *p* = 0.001; see Fig. 2A), as did being younger (β =  − 0.301, t = 7.26; *p* < 0.001) and female (β =  − 0.097, t = 2.22, *p* = 0.027) but not the baseline discount rate (β = 0.08, t = 1.54, *p* = 0.124), learning rate (β = 0.03, t = 0.54, *p* = 0.588), ability to adjust foraging behaviour between the two environments (β =  − 0.07, t = 1.31, *p* = 0.190), or income (β =  − 0.01, t = 0.21, *p* = 0.83).

Further, the overall model predicting depression was significant *F*_(7,385)_ = 8.87, *p* < 0.001, R^2^ = 0.14. Specifically, depression was predicted by greater sensitivity towards reward magnitude (β =  − 0.12, t = 2.42, *p* = 0.016), which remained after correcting for multiple comparisons (*p*_*bonf*_ = 0.032). Depression was also predicted by lower income (β =  − 0.18, t = 2.67, *p* = 0.008, *p*_*bonf*_ = 0.016) and being younger (β =  − 0.27, t = 5.59, *p* = 0.001, *p*_*bonf*_ = 0.002), but not the baseline discounting rate (β = 0.08, t = 1.60, *p* = 0.112), learning rate (β = 0.09, t = 1.77 , *p* = 0.078), ability to adjust foraging behaviour (β =  − 0.01, t = 0.23, *p* = 0.818) or gender (β = 0.04, t = 0.76, *p* = 0.449).

The regression model predicting generalised anxiety was also significant *F*_(7,385)_ = 8.78, *p* < 0.001, R^2^ = 0.14. Generalised anxiety was predicted by a greater sensitivity to reward magnitude (β =  − 0.102, t = 2.11, *p* = 0.035), however this was no longer significant after correcting for multiple comparisons (*p*_*bonf*_ = 0.070). Moreover, generalised anxiety was predicted by being younger (β =  − 0.333, t = 6.86, *p* = 0.001, *p*_*bonf*_ = 0.002) and having lower income (β =  − 0.11, t = 2.29, *p* = 0.023, *p*_*bonf*_ = 0.046). However, baseline discounting rate (β = 0.8, t = 1.62, *p* = 0.106), gender (β = 0.09, t = 1.96, *p* = 0.051), learning rate (β = 0.06, t = 1.29, *p* = 0.199) and ability to adjust foraging behaviour (β = 0.01, t = 0.16, *p* = 0.874) were not significant predictors. See Table 1 for full model results.

**Table 1:** Regression model statistics for each of the outcome variables.

Variable	Social distancing adherence		Active violation of lockdown guidance		COVID-19 anxiety		Generalised anxiety		Depression	
	β	*p*	β	*p*	β	*p*	β	*p*	β	*p*
Age	**0.13**	.009	**−0.14**	.005	**0.30**	< .001	**−0.33**	.001	**−0.27**	.001
Gender	0.06	.224	**−**0.10	.051	**−0.10**	.027	0.09	.051	0.04	.449
Income	**0.11**	.023	**−**0.02	.661	**−**0.01	.830	**−0.11**	.023	**−0.18**	.008
Baseline discounting	**−0.13**	.014	**−**0.02	.570	0.08	.124	0.08	.106	0.08	.112
Magnitude sensitivity	**0.16**	.002	0.08	.099	**−0.17**	.001	**−0.10**	.035	**−0.12**	.016
Learning rate	**−**0.02	.734	**−0.11**	.024	0.03	.588	0.06	.199	0.09	.078
Foraging adjustment	0.04	.409	0.05	.323	**−**0.07	.190	0.01	.499	**−**0.01	.499
*F*	**3.91 (*p* < .001)**		**2.85 (*p* = .007)**		**2.69 (*p* = .01)**		**8.78 (*p* < .001)**		**8.87 (*p* < .001)**	
R^2^	.**07**		**.05**		**.05**		**.14**		**.14**	

Bold indicates p < .050. Gender was coded as male = 1 and female = 0. Analyses that were not pre-registered were adjusted to correct for multiple comparisons.

In an exploratory step, we ran regressions that included an interaction term between the cognitive predictors and COVID-19 anxiety to examine whether mental health symptoms impacted the relationship between task parameters and our measures of lockdown behaviour. This model was significant (*F*_(6,515)_ = 6.67, *p* < 0.001, R^2^ = 0.07) and we found that COVID-19 specific anxiety significantly moderated the relationship between participants’ sensitivity to reward magnitude and adherence to social distancing (β = 0.08, t = 3.38, *p* < 0.001; Fig. 2B). This suggests that as COVID-19 anxiety increased, so did the estimate of magnitude sensitivity on social distancing adherence (i.e., sensitivity towards reward magnitude was a positive predictor of social distancing adherence for individuals with scores of > 50 on the COVID-19 anxiety scale and was a negative predictor of social distancing adherence for individuals with scores of < 50 on the COVID-19 anxiety scale; see Supplementary Fig. 4 for predicted values and fitted lines).

While the model testing whether COVID-19 anxiety moderated the relationship between baseline discounting and social distancing adherence was significant (F_(6, 515)_ = 3.72, *p* < 001, R^2^ = 0.04), we did not find evidence that COVID-19 anxiety moderated the relationship between baseline discounting and social distancing adherence (β = 0.01, t = 0.24, *p* = 0.81). The model testing whether COVID-19 anxiety moderated the relationship between foraging learning rate and active violation behaviour was also significant (*F*_(6,434)_ = 8.67, *p* < 0.001, R^2^ = 0.11). However, we did not find evidence that COVID-19 anxiety moderated the relationship between foraging learning rate and active violation behaviour (β =  − 0.01, t = 1.50, *p* = 0.14).

### Exploratory analysis: cognitive characteristics predict the capability, opportunity and motivation to engage in social distancing

Following a prominent psychological model measuring the capability, opportunity and motivation to enact health behaviours (the COM-B model^26^), we sought to examine whether task parameters predicted individuals’ ability to sustain social distancing across the early acute (T1; see Methods) and established (T2) stages of the pandemic. We first tested whether these parameters predicted appraisals of social distancing at T2, and then examined change in appraisals (T2 − T1; see Supplementary Table 8). Task parameters along with age, gender and income were entered as predictors of self-reported *capability, opportunity* and *motivation* to engage in social distancing.

**Figure 3:**
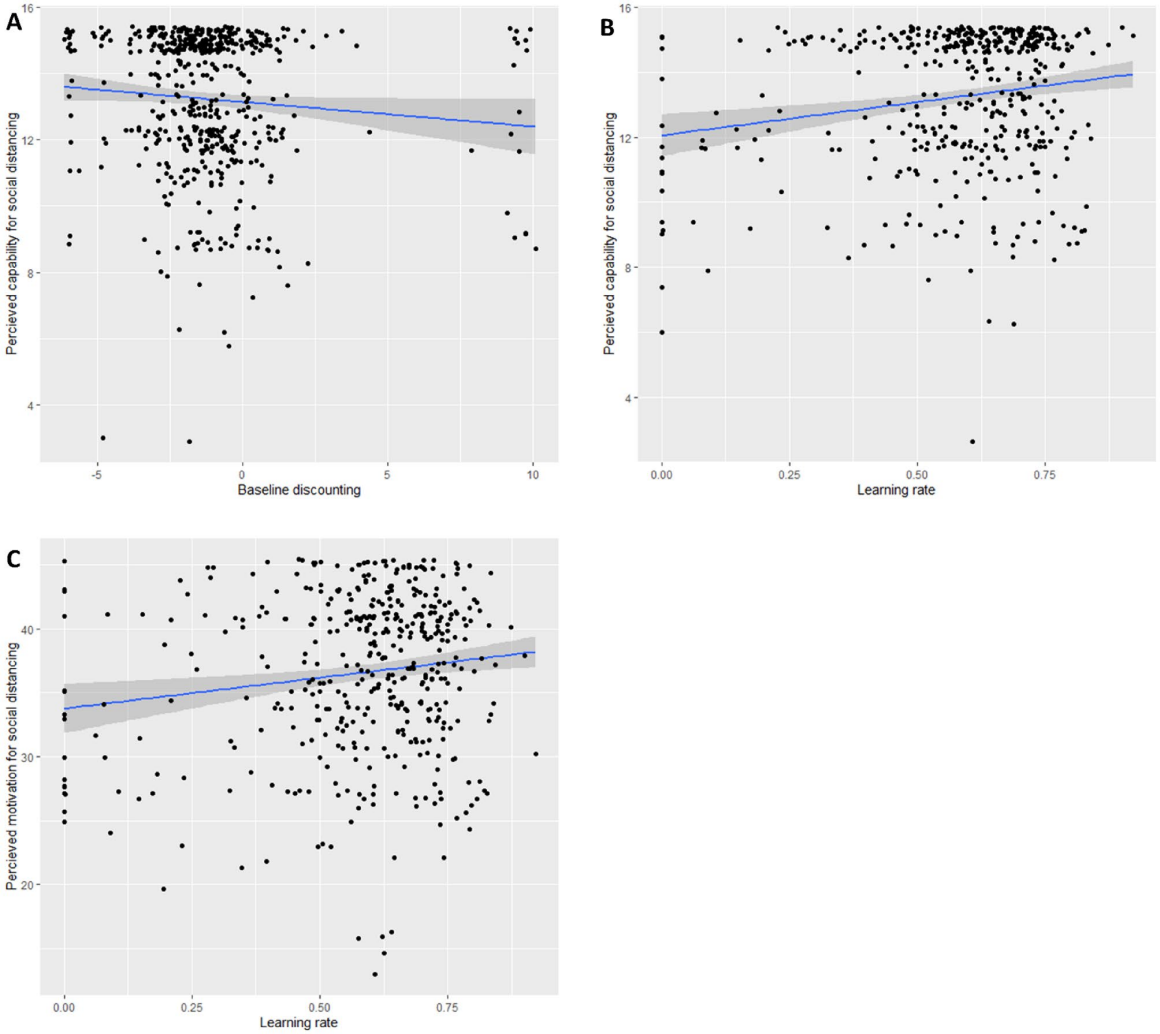
(**A**) Steeper delay discounting predicted higher levels of self-reported capability to enact social distancing during the established phase of the pandemic (T2). (**B**) Lower learning rate predicted lower levels of self-reported capability for social distancing during the established phase of the pandemic (T2). **C)** Higher learning rate also predicted higher levels of self-reported motivation for social distancing. Regression lines are highlighted in blue.

At the established phase of the pandemic (T2), steeper delay discounting, but not magnitude effect (*ps* ≥ 0.666), predicted higher perceived capability (β =  − 0.124, t =  − 2.49, *p* = 0.013, *p*_*bonf*_ = 0.039) and motivation (β =  − 0.074, t =  − 2.35, *p* = 0.019) to enact social distancing, though the motivation subscale was no longer significant after correcting for multiple comparisons (*p*_*bonf*_ = 0.057). Baseline discounting did not predict opportunity (β =  − 0.113, t =  − 2.17, *p* = 0.093) to enact social distancing. Faster learning rate also predicted higher perceived capability (β = 0.19, t = 3.87, *p* = 0.001, *p*_*bonf*_ = 0.003) and motivation (β = 0.14, t = 2.81, *p* = 0.005, *p*_*bonf*_ = 0.015) to enact social distancing independently of delay discounting (see Fig. 3) but learning rate did not predict opportunity to engage in social distancing (β = 0.08, t = 1.65, *p* = 0.297).

**Figure 4:**
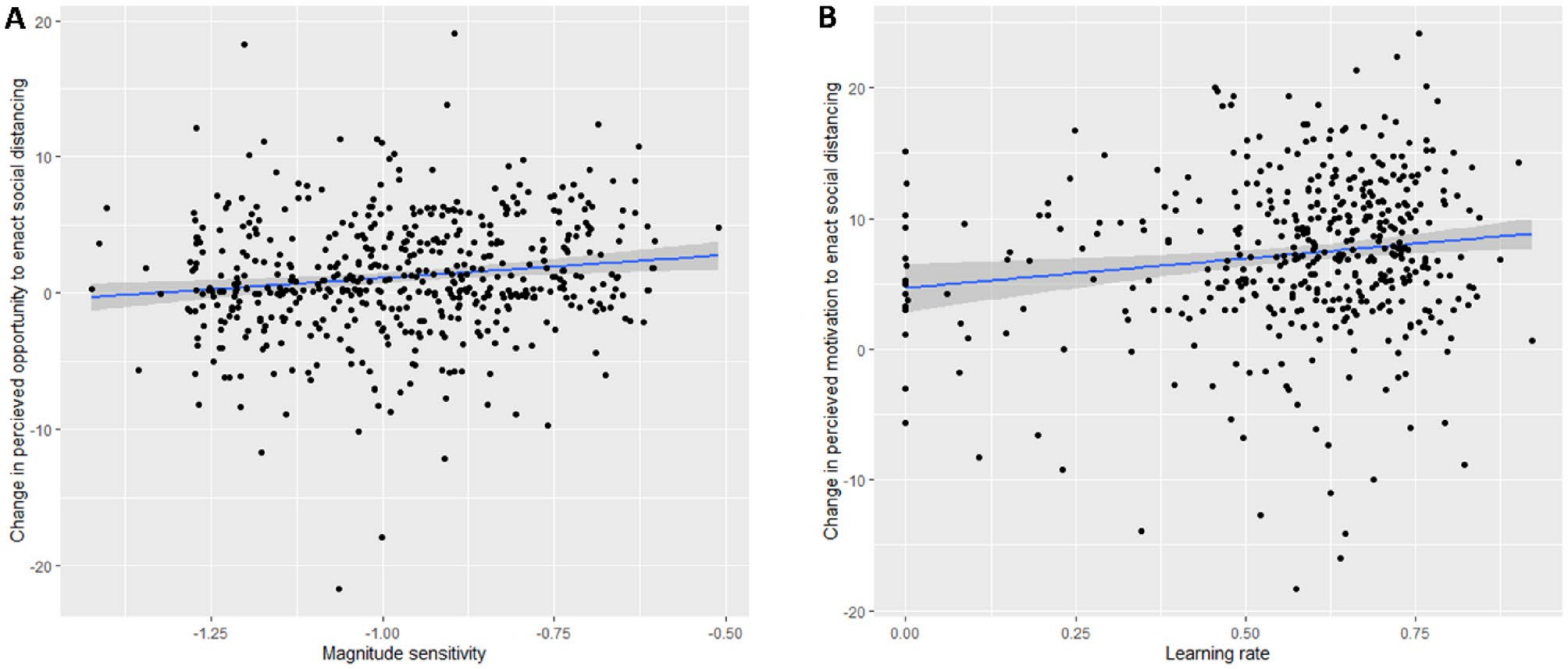
(**A**) Steeper delay discounting magnitude effect (lower values on the x-axis) predicted a greater reduction (T2 − T1) in perceived opportunity to adhere to social distancing. (**B**) Lower learning rate predicted decreased motivation to engage in social distancing. Regression lines are highlighted in blue.

To examine predictors of changes to appraisals of social distancing between the early and established phase of the pandemic, we calculated change scores in the COM-B subscales (T2 − T1). To account for baseline differences, scores at T1 were entered at a subsequent step. Higher sensitivity to reward magnitude, but not delay discounting slope (*ps* ≥ 0.316) predicted a greater reduction in perceived opportunity (β = 0.165, t = 2.93, *p* = 0.004, *p*_*bonf*_ = 0.012; Fig. 4A, but this effect did not remain when additionally accounting for T1 opportunity and correcting for multiple comparisons; *p* = 0.035, *p*_*bonf*_ = 0.105). Higher sensitivity to reward magnitude also predicted perceived capability of social distancing (β = 0.105, t = 1.94, *p* = 0.05, but not when additionally accounting for T1 capability; new *p* = 0.27). In addition, faster learning rate predicted a greater increase in motivation (β = 0.16, t = 3.21, *p* = 0.001, *p*_*bonf*_ = 0.003; Fig. 4B, and remained when additionally accounting for T1 motivation; *p* = 0.004, *p*_*bonf*_ = 0.012) but did not significantly predict greater changes in opportunity (β = 0.09, t = 1.85 , *p* = 0.531) or capability (β = 0.10, t = 1.89, *p* = 0.612).

## Discussion

In the UK and internationally, social distancing and national lockdowns have been key public health policy mandated in response to the COVID-19 pandemic. The present study examined the cognitive predictors of compliance with public health regulations restricting social contact, to direct interventions to maximise uptake of social distancing measures in the population in this and future crises. Our findings demonstrate that both higher levels of baseline delay discounting and greater sensitivity to reward magnitude predicted lower levels of social distance adherence behaviours. In addition, our findings demonstrate that under-valuing recent information in a patch foraging paradigm predicted lockdown violating behaviours. Examining how these cognitive tasks predicted mental health outcomes, we find that people whose delay discounting is more sensitive to the magnitude of reward on offer also showed higher levels of anxiety towards the pandemic. Notably, specific COVID-19 anxiety moderated the effect of delay discounting on social distancing adherence, such that higher levels of anxiety were associated with an increase in the estimated effect of reward magnitude sensitivity on social distancing adherence. Together, these findings suggest that specific features of decision-making make distinct contributions to facilitating adaptive behaviour in response to the COVID-19 restrictions, along with highlighting individuals who may struggle to adjust to these rapid changes.

Adhering to lockdown guidance involves foregoing immediately rewarding activities, such as seeing friends and going to places of leisure, in favour of longer-term goals such as relaxation of restrictions on freedom of movement. Supporting our hypotheses, we found that the degree to which individuals reported adhering to lockdown guidance was predicted by the extent to which they discount delayed rewards. This is consistent with findings that steeper discounters report more problematic health behaviours, such as smoking and alcohol use^8^. The present study extends this to the COVID-19 pandemic, demonstrating the cognitive characteristics associated with compliance to novel public health measures.

The patch foraging data indicated that participants who are quicker to update their beliefs about environments were less likely to violate lockdown measures. Rapidly updating one’s beliefs in response to new information is an adaptive decision-making strategy in conditions of volatility, where the associations between actions and outcomes are rapidly changing^7^. The COVID-19 pandemic has been met with rapid policy changes in public health guidelines to slow the spread of the virus, which has required frequent behavioural changes across the population. Individuals who prioritise recent information may therefore demonstrate better adaption to novel action-outcome contingencies, allowing them to uptake behaviours with greater ease. In contrast, individuals who update their beliefs more slowly may have acted on outdated pre-pandemic information, thereby engaging in behaviours that were in violation of lockdown measures. This interpretation was supported by our analyses of participants’ self-reported capability, opportunity, and motivation to engage in social distancing, as faster updating of beliefs predicted capability and motivation to engage in social distancing measures at the established phase of the pandemic.

Results of the factor analysis indicated that adherence to social distancing and active violation of lockdown measures may be distinct behaviours. The active violation factor utilised in the present study included items that refer to behaviours that were permissible prior to the implementation of the lockdown measures (e.g., gathering in groups). In contrast, lockdown adhering items refer to behavioural modifications that were required in response to the virus (e.g., remaining at least 2 m away from others). This provides some insight into why the cognitive tasks predicted each scale separately; individuals engaging in lockdown violating behaviours were those who under-weigh recent information and may therefore be basing their behaviour on pre-lockdown information. Importantly, this suggests that individuals who actively violated lockdown measures may not purposefully engage in rule infractions, but rather may not have been acting on recent information that had been disseminated regarding the lockdown measures.

With regards to mental health, our findings demonstrate that the extent to which people’s intertemporal decisions are sensitive towards the magnitude of reward on offer, but not their baseline discounting rate, predicted high levels of specific COVID-19 anxiety. We predicted that people who more steeply discount these delayed future rewards are likely to be those who experience greater levels of distress from “involuntary” lockdown; yet, we found instead that distress was predicted by the extent to which they were sensitive to the magnitude of reward on offer. There is evidence that sensitivity to reward magnitude is influenced by self-control, specifically the recruitment of cognitive resources during decision-making^18^. One possibility is that those who are more sensitive to reward magnitude may struggle to enact social distancing because of a reduced capability to direct cognitive resources towards implementing this behaviour. Whilst speculative, this would be consistent with our finding that these participants reported a greater reduction in perceived opportunity to enact social distancing, as measured by the COM-B scale.

We also found that anxiety moderated the relationship between participants’ sensitivity to reward magnitude and their social distancing adherence. For individuals who scored above the midpoint of our scale for COVID-19 anxiety, less sensitivity towards the magnitude of rewards was associated with greater adherence to social distancing. In contrast, for individuals scoring below the midpoint of our COVID-19 anxiety scale we found the reverse pattern, namely that *greater* sensitivity to reward magnitude was associated with greater adherence to social distancing. As such, whether sensitivity to reward magnitude was a positive or negative predictor of social distancing adherence differed according to the levels of anxiety participants reported towards the COVID-19 pandemic. One tentative interpretation of this finding is that, for individuals with lower COVID-19 anxiety, a factor involved in these participants’ compliance with social distancing is their sensitivity towards larger delayed rewards that would be attained through adhering to social distancing (for example, arising from expediting the resolution of the pandemic). Future work could examine the perceived costs and benefits motivating social distancing compliance to test this possibility.

The findings of the present study may have implications for public health bodies seeking to increase compliance with social distancing, as there is evidence that delay discounting is amenable to intervention^27,28^. For example, episodic future thinking can reduce delay discounting, and this decline in discounting has been associated with a reduction in harmful health behaviours, such as cigarette smoking and fast-food consumption^29,30^. Whether such interventions can be translated to the COVID-19 pandemic to increase compliance with social distancing measures will be an important avenue for future research. However, it is important to note that the effect sizes for both tasks were relatively small, meaning the processes measured by these tasks may only make a small contribution to individuals’ compliance with social distancing, whereas other variables (particularly age) appear to play a larger role.

It is also possible that additionally measuring other relevant cognitive processes, such as discounting in the loss domain (not quantified in the current study) could yield stronger predictive value for social distancing compliance^31^. A related possibility is that discounting of social rewards^32^ is another mechanism involved in social distancing that is dissociable from monetary discounting^33,34^, although see^35,36^. With regards to the learning rate, separating this parameter for better-than-expected and poorer-than-expected outcomes^37^ may also increase the predictive value of this task, as the COVID-19 pandemic has been associated with adjusting to negative changes in the environment^38^. As such, individuals who have a higher learning rate for negative outcomes may be better equipped to follow social distancing guidelines.

There are some important limitations to consider with the present study. The two-factor solution may be counterintuitive as adhering to social distancing and actively violating lockdown measures could be considered the inverse of one another. However, we note that this same two-factor solution has also been found in another, larger sample of UK adults (Gibson-Miller et al., in prep). Further, cognitive task-based measures were only collected at one timepoint, meaning we were unable to examine whether these variables changed over the course of the pandemic. Some variables, such as anxiety, were initially high after the nationwide lockdown was imposed and slowly stabilised in the following months^39^. Given the debate about whether delay discounting is influenced by state as well as being a stable trait^9^, we cannot rule out that behaviour on our cognitive measures may have differed if we had been able to additionally measure them at the first wave time point. This limits the interpretations we can draw about the relationships between these measures and the wave one COM-B data. Finally, while the Kirby discounting task we utilised in the present study has sensitivity to a broad range of discount rates, it’s restricted range of reward magnitudes limits its precision in estimating participants’ sensitivity towards the magnitude effect. Future research could utilise a measure with a wider range of reward magnitudes to increase the precision of this measure.

In conclusion, the present study aimed to examine the cognitive predictors of social distancing and poorer mental health outcomes in a representative UK sample. Our findings demonstrated that steeper delay discounting was associated with less adherence to social distancing, whereas undervaluing recent information was associated with higher rates of lockdown violations. Notably, our findings provide empirical evidence that social distancing adherence and active violation of lockdown measures are two distinct dimensions, which are predicted by separate cognitive processes. These findings can guide future research that may seek to leverage cognitive interventions to maximise public adherence to social distancing and minimise violations of these measures. Further, our findings indicate that individuals who are more sensitive to reward magnitude in delay discounting experienced poor mental health symptoms as a result of lockdown measures. This highlights the need to provide opportunities for these individuals to attain immediate sources of reward and mitigate anxiety that results from these novel policies. Overall, these findings provide insight into the cognitive factors that predict adaption to the public health measures introduced to reduce the spread of the COVID-19 and highlight the potential of cognitive science to inform our understanding of behaviour during the pandemic.

## Methods

### Participants

A total of 442 participants completed both tasks. There was a wide age range (18 to 83 years; *M* = 52.7, *SD* = 14.9) with 63.1% (279 participants) of the sample being male, 36.9% were female (163 participants) and 1.3% (six participants) did not reporting their gender. Annual household income levels were varied, with 17% earning under £15,491, 16.7% earning between £15,491–£25,340, 17.6% earning between £25,341-£38,740, 22.8% earning between £38,741–£57,930 and 24.6% earning above £57,930.

We recruited participants from a longitudinal study examining the psychological impact of the COVID-19 pandemic in the UK (the COVID-19 Psychological Research Consortium (C19PRC) Study; see^40^, for a complete description of the methodology). This cohort is a large, representative sample of UK adults collected through Qualtrics (*N* = 2878; quota sampling methods ensured that the sample was representative in terms of age, sex, and gross household income). A total of 1406 participants from this cohort had completed both the first (early acute phase of pandemic) and second (established phase) waves of the C19PRC study. A subsample of 442 of these individuals subsequently completed the cognitive-based tasks reported here. Ethical approval for the cognitive tasks was received from University College London ethics committee and all methods were carried out in accordance with relevant guidelines and regulations.

Data collection took place immediately after the second wave of this survey (28th April 2020 to 10th May 2020), to facilitate utilising responses as part of that battery. After providing informed consent, participants completed the tasks on www.gorilla.sc, an online platform for remotely conducting cognitive science studies. Our hypotheses were pre-registered before data collection was completed (delay discounting: https://osf.io/r89pf, patch foraging: https://osf.io/maqxd). Note, the hypotheses were reordered in the manuscript to improve readability. The correspondence between the pre-registered hypotheses and those reported in the manuscript are detailed in the Supplementary Material. Two of the three hypotheses in each pre-registration are tested here; a third hypothesis in each concerning the prediction of over-purchasing behaviour will be examined in a separate paper.

### Measures

*Delay Discounting:* Delay discounting was quantified using a widely used 27-item measure^6^. On each trial, participants are presented with a hypothetical choice between a sum of money available immediately and a larger sum for which they must wait (varying between 7 and 186 days). For example, participants were asked if they would prefer £26 now or £30 in 15 days. The model (hyperbolic discounting with magnitude effect)^41^ fits were good, predicting a median of 96.3% (SD = 0.06) of choices. The model was able to capture variability in delay discounting across participants, and the discount rate varied as a function of reward magnitude. The group level parameter estimates and parameters for individual participants are reported in the Supplementary Materials (see Supplementary Figs. 1 and 2).

**Figure 5:**
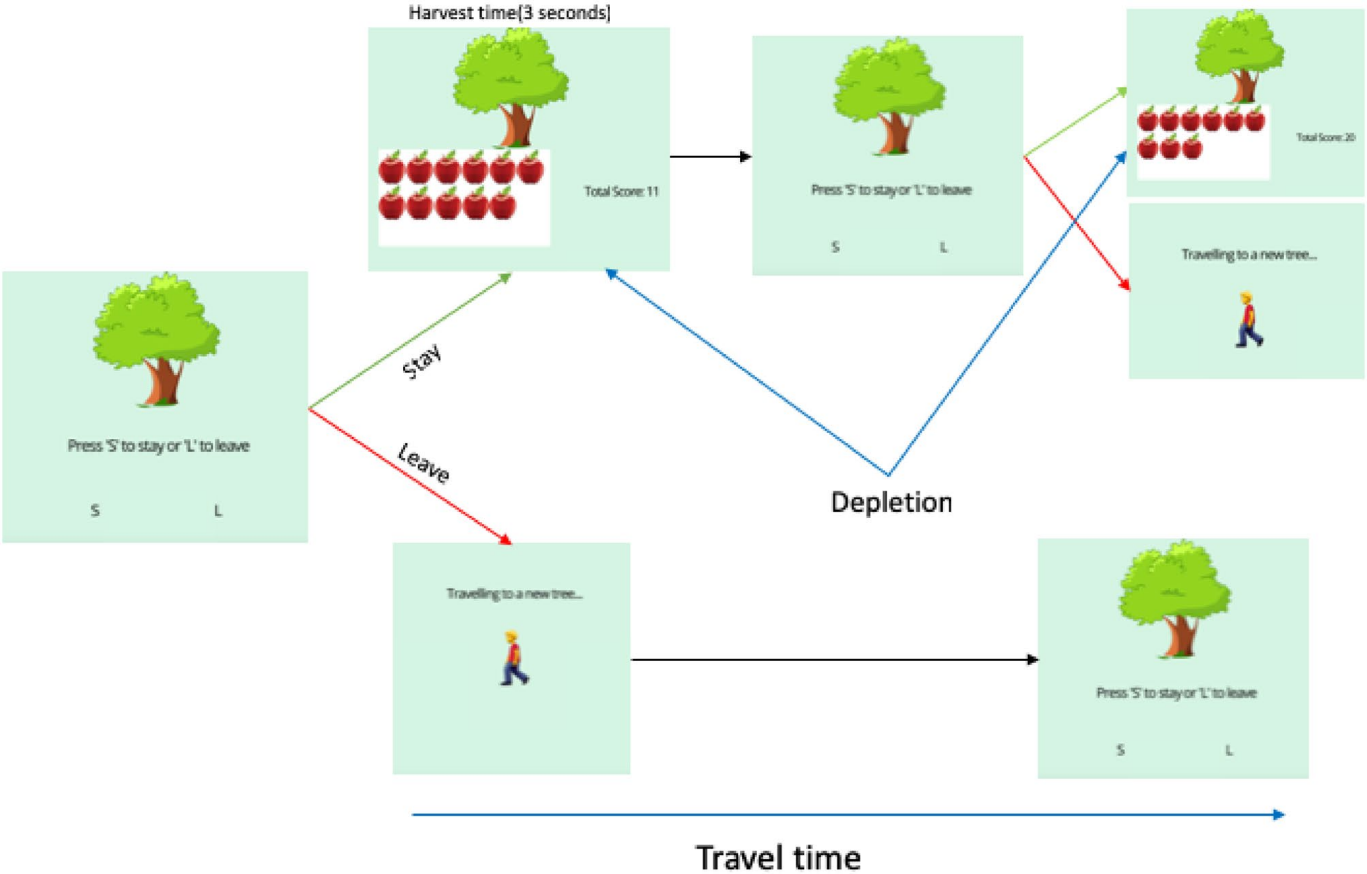
Outline of the patch foraging task. Participants who chose to stay (top panel) are presented with the number of apples they collected on that trial before being returned to the tree. Participants who chose to leave (bottom panel) are presented with a screen for six seconds before arriving at a new patch.

*Patch Foraging:* Patch foraging was quantified in a decision-making task requiring participants to evaluate the trade-off between exploiting a current resource for a gradually diminishing number of rewards versus exploring a novel resource with a fresh distribution of rewards. We used an apple picking paradigm, with participants instructed to collect as many apples as possible within the time limit^24^. Trees represented individual patches that participants could forage by choosing to ‘stay’ to collect rewards (apples; see Fig. 5). The longer participants chose to remain with a single patch, the fewer rewards would be harvested on each stay decision until harvesting the patch yielded zero rewards. Participants completed two unique environments or ‘orchards’, which differed in the number of rewards available. The availability of rewards was manipulated through changing the rate at which apples depleted from patches, which was based on previous research^42^. Each environment was presented for five minutes with the order of presentation counterbalanced across participants.

Consistent with previous research, participants adjusted the point that they left patches according to the richness of the environment^32,42^. Specifically, participants explored more in the richer environment and explored less in the poorer environment. This was confirmed with a paired t-test *t*(447) = 5.08, *p* < 0.001, with participants having a higher leaving threshold in the rich environment (*M* = 5.15, *SD* = 2.35) compared to the poor quality environment (*M* = 4.72, *SD* = 2.26).

*Mood and anxiety symptoms*: Analyses included an item probing specific anxiety around COVID-19 used in previous research^2,40^. The wording of the item was: “How anxious are you about the coronavirus COVID-19 pandemic?” and participants gave slider ratings on a 0–100 scale. In addition, we measured general anxiety symptoms using the Generalized Anxiety Disorder questionnaire (GAD-7; ^43^), with total scores ranging from 0–21. Scores higher than 15 indicate severe anxiety. Finally, depression was measured using the Patient Health Questionnaire depression scale (PHQ-9; ^44^), with total scores ranging from 0–27. Scores equal to or greater than 20 indicate severe depression.

*COVID-19 risk behaviours:* Participants completed 12 items that probed behaviours related to guidance given by the UK Government to reduce the risk of infection by coronavirus. Participants were asked: “In the past week, to reduce your risk of being infected by or passing on the coronavirus to others, on how many days of the week have you” and included items such as: “Stayed at least 2 m (6ft) away from others when in public”. Some items were reverse scored, such as: “Gathered in a group of more than two people in a park or other public space”, and “Engaged in close contact greetings”. Likert scale responses were collected ranging from: “Not at all” (0), “1–2 days a week” (1), “3–4 days a week” (2), “Most days” (3), and “Every day” (4).

Participants additionally completed 18 items assessing psychological inclination to comply with social distancing, in line with a theoretical model of health behaviour that measures 'capability', 'opportunity' and 'motivation' (COM-B model^26^). These are reported in greater detail elsewhere^45^ but included items measuring beliefs about social distancing rules (e.g. “staying in your home most of the time, exercising outside once a day, not meeting up with friends and relatives, maintaining a 2-m distance from people, working from home”) during the COVID-19 pandemic. These measures were collected during both the first and second waves of the C19PRC study^40,45^. Comparable measures of COVID-19 risk behaviours were not collected during the first wave of the C19PRC study.

### Planned analyses

Delay discounting was quantified using a hierarchical Bayesian model implemented in a widely available analysis toolbox^41^. Because the discount rate is known to decrease as the magnitudes of rewards increases (the magnitude effect), we modelled log discount rates as a linear function of log reward magnitude. Empirically, studies examining the magnitude effect show that log discount rate decreases as a linear function of log reward magnitude^46^. Data from multiple studies are well fit by a linear function in log reward, log discount space^41^. We therefore obtained estimates of this slope (*m*) and intercept (*c*)—lower values of *m* correspond to greater sensitivity towards magnitude effects and lower values of *c* correspond to lower baseline discount rates for a given reward magnitude. To capture both the slope and intercept of the delay discounting function, both parameters (m and c) were entered as predictors in regression analyses, along with patch foraging parameters (below). Separate analyses tested our hypotheses that these parameters would predict mood symptoms (generalised anxiety, depression, and specific COVID-19 anxiety) and social distancing behaviour.

We utilised two variables from the patch foraging task. The first, learning rate, was estimated from an established computational model of learning in the present foraging paradigm^21^. The model is derived from Marginal Value Theorem, which describes optimal foraging behaviour^47^. The equation states that the decision-maker should leave their current patch when the rewards expected from harvesting the patch fall below the average reward intake for that environment. As participants do not know the average reward rate a priori, they infer this over time, which can be expressed by the equation ^24^: ${p}_{i}={\left(1-\alpha \right)}^{Ti}\frac{{s}_{i}}{{T}_{i}}+\left(1-{\left(1-\alpha \right)}^{Ti}\right){p}_{i-1}$ (see Supplementary Table 1 for a list of notations). Learning rate is quantified by the free parameter ${\upalpha }$. As this is formulated as the complement of traditional learning rates, we then subtracted participants’ learning rate from 1 to transform this value into the conventional parameterisation of this variable. Therefore, higher values on this parameter indicate that participants weighted recent information higher. The alpha parameter was averaged across the two foraging environments, as this did not significantly change between the two conditions *t*(398) = 1.56, *p* = 0.116.

To demonstrate that the model yielded similar parameter estimates from data where the parameters were known, we simulated data with alpha levels between 0–1 in increments of 0.001 and ran the model using this simulated data. Results of a Pearson’s correlation analysis suggested a strong positive correlation between simulated parameters and those estimated by the model (*r* (999) = 0.94, *p* < 0.001, 95% CI [0.93, 0.94]), which demonstrated that the parameter was recoverable.

The second variable was how well participants were able to adjust their behaviour according to the statistics of the two foraging environments. To derive this variable, we first calculated how much participants deviated from the optimal leaving threshold (according to Marginal Value Theorem) in each environment (see ^42^), which provided a standardised metric of optimal foraging. The difference between these deviations was subsequently calculated, with higher values indicating participants were less successful at adjusting their behaviour to the structure of different environments.

As the social distancing scale was developed for the purposes of this study and is being validated in parallel (Gibson-Miller et al., in prep), we conducted a factor analysis to examine whether participants’ self-reported behaviours were explained by a single latent variable. First, we determined the number of factors using parallel analysis, which suggested a two-factor solution. We then conducted an exploratory factor analysis using an oblique rotation, which suggested the two-factor solution was sufficient. Finally, these items were entered into a confirmatory factor analysis using the lavaan package for R^48^. Goodness of fit indices suggested that a two-factor solution was a good fit to the data (χ^2^ = 13.36, *p* = 0.646, RMSEA < 0.001). Full results of the factor analysis are detailed in the Supplementary Material.

In this model, one factor represented adhering behaviours (e.g., “Stayed at least 2 m away from others in public”), which higher scores denoting more behaviours that were consistent with adhering to social distancing guidelines. The second factor represented behaviours that actively violated lockdown measures (e.g., “Gathered in a group of people”). The active violation subscale was not normally distributed, and we therefore conducted a Box-Cox transformation using the MASS package^49,50^. Higher values on this scale indicate the participant reported engaging these behaviours more frequently. For details on factor loadings, see the Supplementary Material. The social distancing adherence and active violation scales generated from the confirmatory factor analysis were subsequently utilised as outcome measures.

## Supplementary Information

Supplementary Information.
