# Supplementary material for: Delay discounting and under-valuing of recent information predict poorer adherence to social distancing measures during the COVID-19 pandemic
Source: Sci Rep. 2021 Sep 28;11:19237. doi: 10.1038/s41598-021-98772-5 (PMC8479072; doi:10.1038/s41598-021-98772-5)
Supplement: Supplementary file 1 — Supplementary Information. [file 41598_2021_98772_MOESM1_ESM.docx]

**Supplementary Material: Delay discounting and under-valuing of recent information predict poorer adherence to social distancing measures during the COVID-19 pandemic**

**Supplementary Introduction**

We had two additional pre-registered hypotheses, which were not reported in the main text: H3) Higher levels of trait catastrophising will predict greater discounting and H4) Participants who report higher COVID-19 anxiety will remain with patches for longer (i.e. will have a lower leaving threshold) whereas those reporting less COVID-19 anxiety will remain with patches for less time (i.e. will have a higher leaving threshold). Additional hypotheses included in this pre-registration document will be examined in another manuscript. The preregistered hypotheses and their ordering reported in the manuscript are reported in Supplementary Table 1.

Supplementary Table 1: Correspondence between the hypotheses in the pre-registration document and in the manuscript.

| **Hypothesis** | **Pre-registration document** | **Number in the pre-registration** | **Number in the manuscript** |
| --- | --- | --- | --- |
| Higher levels of delay discounting will predict poorer adherence to social distancing. | Delay discounting: <https://osf.io/r89pf> | H2a | H1a |
| Higher levels of discounting will predict *greater* adherence to social distancing | Delay discounting: <https://osf.io/r89pf> | H2b | H1a |
| Higher learning rates would predict greater social distancing adherence | Patch foraging: <https://osf.io/maqxd> | H1 | H1b |
| Poorer mental health, including specific anxiety about the COVID-19 pandemic, would be predicted by higher levels of delay discounting | Delay discounting: <https://osf.io/r89pf> | H1b | H2a |
| Poorer mental health, including specific anxiety about the COVID-19 pandemic, would be predicted by a reduced ability to adapt the point at which they left patches between foraging environments | Patch foraging: <https://osf.io/maqxd> | H2b | H2b |
| Higher levels of trait catastrophising will predict greater discounting | Delay discounting: <https://osf.io/r89pf> | H1a | H3 |
| Participants who report higher COVID-19 anxiety will remain with patches for longer (i.e. will have a lower leaving threshold) whereas those reporting less COVID-19 anxiety will remain with patches for less time (i.e. will have a higher leaving threshold). | Patch foraging: <https://osf.io/maqxd> | H2a | H4 |

**Supplementary Methods**

*Delay Discounting:* See Supplementary Figure 1 for parameters for individual participants.


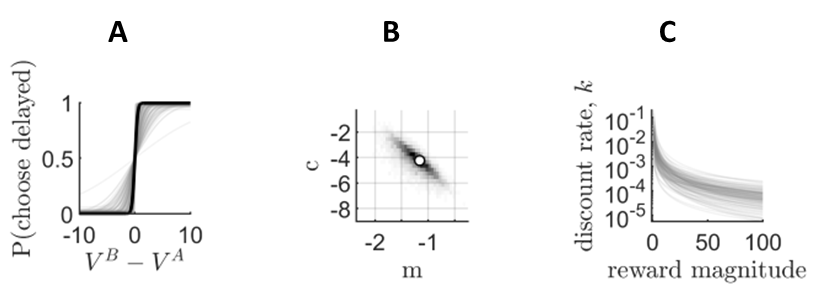


Supplementary Figure 1. Group level parameter estimates for delay discounting. **A)** The probability of choosing option A or B as a function of the difference in inferred subjective reward (via hyperbolic discounting); the black line represents the posterior mean, and grey lines represent samples from the posterior distribution. **B)** The posterior distribution of delay discounting magnitude effect slope (m) and intercept (c); see planned analyses for a description of these parameters; **C)** The magnitude effect represented by draws from the posterior distribution of m and c.


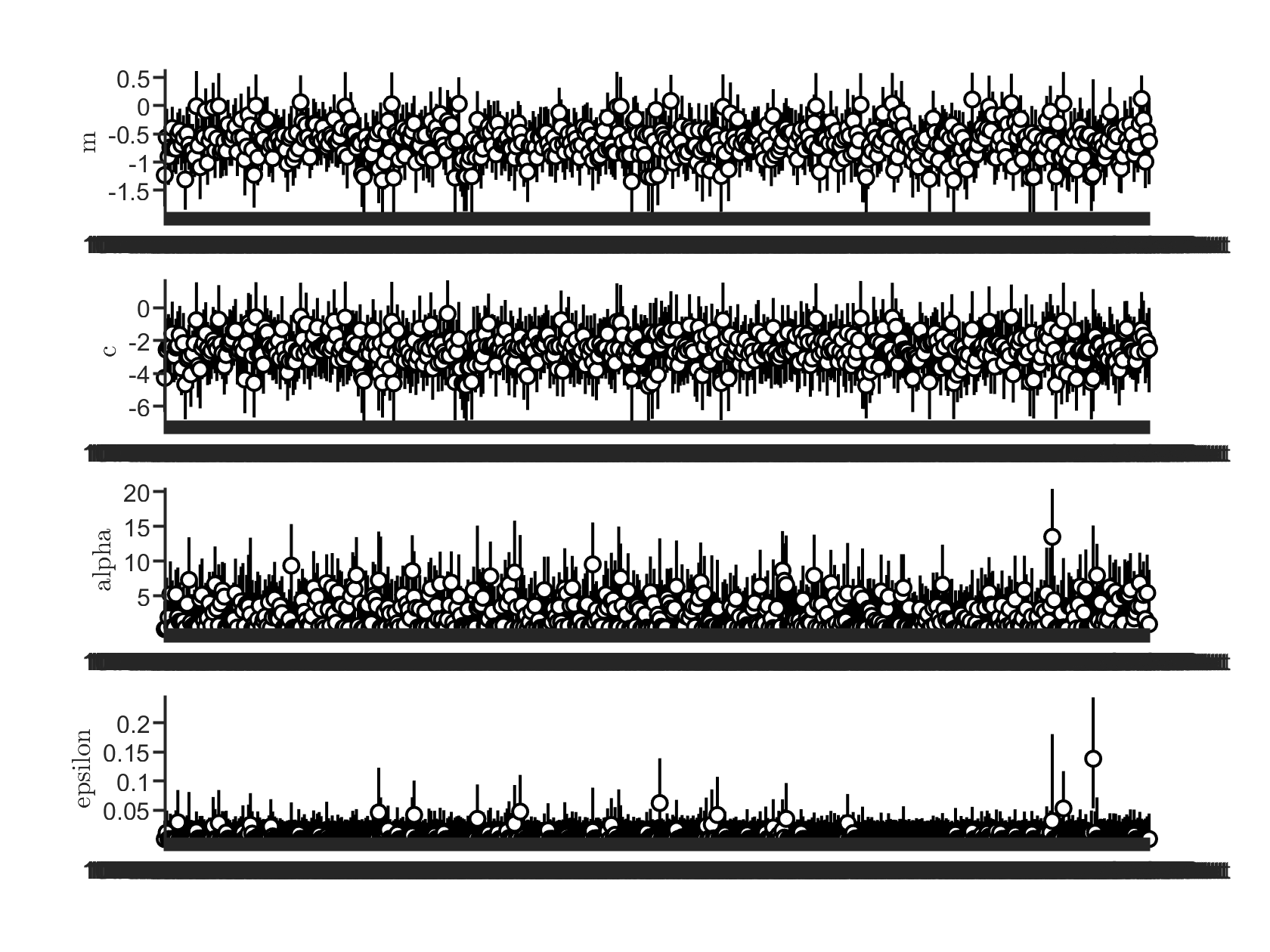


Supplementary Figure 2. Parameters extracted for individual participants: delay discounting slope (m), intercept (c), acuity (alpha) and error (epsilon).

*Patch foraging task:* Each decision to stay took three seconds in total, including the choice time and the presentation of the apples harvested. Alternatively, participants could choose to leave the current patch to explore a novel tree, which would incur a time delay of six seconds. Each new patch had a fresh distribution of rewards, which was drawn from a Gaussian distribution with a mean of 10 (SD = 1). The richness of each environment was manipulated through changing the rate at which rewards depleted within patches. Participants should adjust the point at which they leave patches (known as their leaving threshold) according to the richness of the environment, exploring more in environments where nearby patches are rich in rewards and exploring less in environments where nearby patches are poorer in quality. Results demonstrated that participants adjusted their leaving threshold in a manner consistent with optimal foraging theory (see Supplementary Figure 3). Participants’ leaving threshold in the two foraging environments were utilised as additional outcome variables to those mentioned in the main text and was excluded from these analyses due to the high correlation with the learning rate parameter (see Supplementary Table 5). Higher leaving thresholds indicate that participants explored more often.


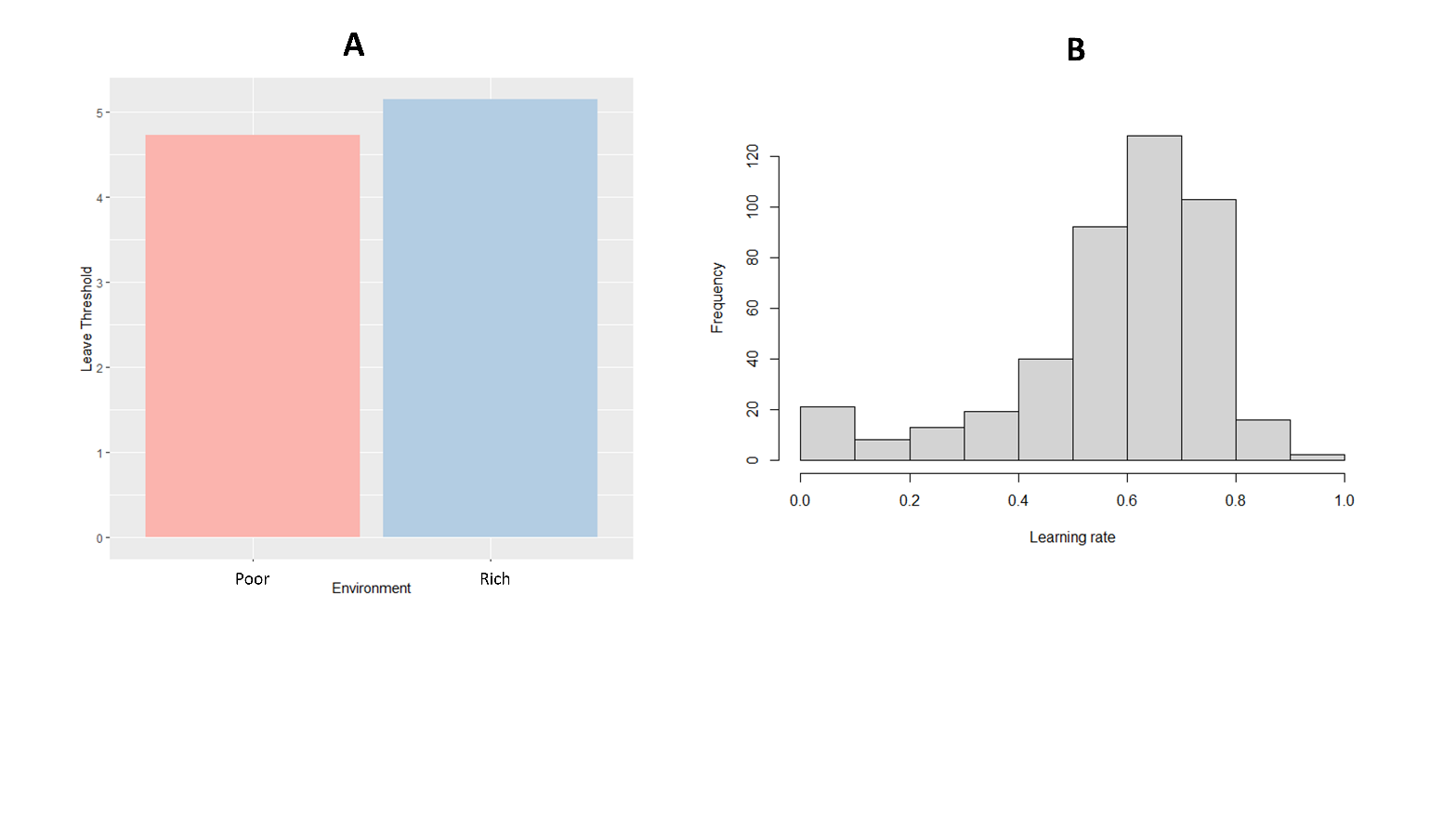


Supplementary Figure 3: A) Plot demonstrating participants' leaving threshold (y-axis) for both the poor and rich foraging environments (x-axis). Higher leaving thresholds indicate participants explored more. B) Plot demonstrating the distribution of scores for the learning rate parameter.

Supplementary Table 2. MVT Learning equation notation adapted from Constantino & Daw (2015)

| Notation | Definition |
| --- | --- |
| *T_i_* | Time cost associated with the decision |
| *S_i_* | Last observed reward state of the current patch |
| *α* | Learning rate |
| *p_i_* | Average reward rate as estimated by the agent |

*Social distancing adherence and lockdown violation scales*

Several items from the original questionnaire were excluded from the factor analyses due to issues that were identified after data collection. These were: “Left the house to care for a vulnerable person”, as only 31.8% of the sample reported knowing a vulnerable individual; “Worked from home”, as only 61.1% of the sample was employed; “Reminded children about social distancing rules”, as only 24.2% of the sample reported having children.

To determine the number of factors that best explained the data, we first calculated the eigenvalues of each factor. This suggested a two-factor solution, as two factors had eigenvalues above one. The values were as follows: factor 1 – 3.75, factor 2 – 1.62, factor 3 - 0.76 factor 4 - 0.68, factor 5 - 0.49, factor 6 - 0.31, factor 7 - 0.24, factor 8 - 0.15. Using parallel analysis, which utilises scree plots to determine the number of factors, we also found that a two-factor solution was the best fit to the data.

We then ran exploratory factor analysis using an oblique rotation on the correlation matrix to identify the factor loadings for each item. The EFA confirmed that a two-factor solution was sufficient. The individual factor loadings can be found in Supplementary Table 3.

Supplementary Table 3. Standardized loadings (pattern matrix) based upon correlation matrix

| Item | Factor 1 – Social Distancing Adherence | Factor 2 – Lockdown Violation |
| --- | --- | --- |
| 1 | 0.6 | -0.17 |
| 2 | 0.75 | 0.06 |
| 3 | 0.35 | 0.23 |
| 4 | 0.26 | 0.48 |
| 5 | 0.02 | 0.86 |
| 6 | -0.04 | 0.82 |
| 7 | 0.04 | 0.86 |
| 8 | -0.05 | 0.95 |

This factor structure was then confirmed using a confirmatory factor analysis (CFA). While twelve items were measured in total, only eight of these loaded onto the scales as indicated by the CFA. The remaining items were excluded from further analysis. The final factor loadings can be found in Supplementary Table 4.

Supplementary Table 4. Social distancing items and factor loadings.

| Items |  | Standardized Loading | Standard Errors |
| --- | --- | --- | --- |
|  | *Factor 1 - Social Distancing Adherence Behaviours* |  |  |
| 1 | Left the house for food, health reasons, or work | 0.34*** | 0.09 |
| 2 | Stayed at least 2 metres away from others in public | 1*** | 0.00 |
| 3 | Exercised outside once a day | 0.75*** | 0.17 |
| 4 | Exercised outside more than once a day | 0.31*** | 0.09 |
|  |  |  |  |
|  | *Factor 2 - Lockdown Violating Behaviours* |  |  |
| 1 | Left the house for food, health reasons, or work | -0.55*** | 0.15 |
| 4 | Exercised outside more than once a day | -1.03*** | 0.27 |
| 5 | Met up with friends | -1.58*** | 0.38 |
| 6 | Gathered in a group of people | -1.67*** | 0.40 |
| 7 | Driven to a green space to exercise | -1.57*** | 0.38 |
| 8 | Engaged in close contact greetings | -1.54*** | 0.36 |

*** *p* <.001

**Supplementary Results**

A paired sample t-test was conducted on the learning rate between the two foraging environments. There was not a significant difference between participants’ learning rate in the rich and poor foraging environments t(398) = 1.56, *p* = .116.. As such, we averaged learning rate across the two environments in subsequent analyses. We did not observe any correlations between patch foraging and delay discounting parameters (see Supplementary Table 4).

Supplementary Table 5: Correlation matrix of the delay discounting and patch foraging variables. Correlation analyses were two-tailed.

| Pearson's Correlations | | | | | | | | | | | | | | | | |  |
| --- | --- | --- | --- | --- | --- | --- | --- | --- | --- | --- | --- | --- | --- | --- | --- | --- | --- |
| Variable | |  | | Learning rate | Leaving threshold | | | Deviation change | | | m_mode | | | c_mode | | |  |
| Learning rate |  | Pearson's r |  | — |  |  |  | |  |  | |  |  | |  |  | |
|  |  | p-value |  | — |  |  |  | |  |  | |  |  | |  |  | |
| Leaving threshold |  | Pearson's r |  | 0.828 |  | — |  | |  |  | |  |  | |  |  | |
|  |  | p-value |  | < .001 |  | — |  | |  |  | |  |  | |  |  | |
| Deviation change |  | Pearson's r |  | -0.125 |  | 0.013 |  | | — |  | |  |  | |  |  | |
|  |  | p-value |  | 0.013 |  | 0.802 |  | | — |  | |  |  | |  |  | |
| m_mode |  | Pearson's r |  | -0.032 |  | -0.008 |  | | 0.001 |  | | — |  | |  |  | |
|  |  | p-value |  | 0.507 |  | 0.871 |  | | 0.977 |  | | — |  | |  |  | |
| c_mode |  | Pearson's r |  | -0.014 |  | 0.026 |  | | 0.028 |  | | 0.204 |  | | — |  | |
|  |  | p-value |  | 0.769 |  | 0.587 |  | | 0.583 |  | | < .001 |  | | — |  | |
|  | | | | | | | | | | | | | | | | |  |

**Cognitive characteristics predicted adherence to social distancing during lockdown**

As the active violation subscale was not normally distributed, we inspected the data and observed that the variance was substantially larger than the mean, which indicates overdispersion. As such, we ran a quasipoisson regression, which is suited to data that is not normally distributed and is overdispersed^1^. Using the untransformed lockdown violation scale as the outcome variable, the results of this analysis were consistent with our OLS regression, as participants’ learning rate was a significant predictor of their violation of lockdown guidance as was being younger (see Supplementary Table 6 for a comparison of the results from the OLS and Poisson regressions).

Supplementary Table 6: Regression model statistics for the active lockdown violation scale. Note, the OLS model uses the Box-Cox transformed variable as the outcome variable whereas the quasipoisson model uses the untransformed variable as the outcome variable.

| Variable | OLS Model | | Quasipoisson Model | |
| --- | --- | --- | --- | --- |
|  | β | *p* | β | *p* |
| Age | -0.14 | .005 | -0.20 | <.001 |
| Gender | -0.10 | .051 | -0.9 | .072 |
| Income | -0.02 | .661 | 0.02 | .747 |
| Baseline discounting | -0.02 | .570 | -0.03 | .527 |
| Magnitude sensitivity | 0.08 | .099 | 0.08 | .109 |
| Learning rate | -0.11 | .024 | -0.11 | .023 |
| Foraging adjustment | 0.05 | .323 | 0.07 | .141 |

**Delay discounting but not leaving threshold predicted mood and anxiety symptoms**

Sensitivity to reward magnitude during delay discounting remained a strong predictor of specific COVID-19 anxiety (p = .007) when additionally accounting for generalised anxiety symptoms, indicating independent relationships of delay discounting with specific and general anxiety symptoms. This suggests that delay discounting is associated with state anxiety symptoms secondary to the COVID-19 pandemic, rather than a trait propensity towards anxiety.

To examine the contribution of delay discounting parameters on anxiety and mood symptoms, we first modelled anxiety and mood symptoms as a function of the delay discounting parameters. In a separate step, we then entered age, gender and income as control variables to examine changes in the estimates in the presence of these covariates. The estimates for the delay discounting parameters with and without control variables are presented in Supplementary Table 7 below.

Supplementary Table 7. Estimates for the delay discounting magnitude effect with and without control variables entered.

|  | Estimates without control variables added | Estimates with control variables added |
| --- | --- | --- |
| Generalised anxiety (GAD-7) | β = -.12, t = -2.25, *p* = .025 | β = -.102, t = 2.11, *p* = .035 |
| COVID-19 Anxiety | β = -.18 t = -3.32, *p* < .001 | β = -.17, t = 3.34, *p* = .001 |
| Depression (PHQ-9) | β = -.10, t = -1.88, *p* = .061 | β = -.12, t = 2.42, *p* = .016 |

A regression analysis was conducted to examine whether higher levels of trait catastrophising predicted greater delay discounting. The overall model was not significant (*F*_(5,393)_ = 0.07, *p* = .785, R^2^ < .01 and we did not find evidence that trait catastrophising was not a significant predictor of delay discounting (β= -0.01, t = .27, *p* = .785).

Finally, to examine whether there was any association between leaving threshold in the two foraging environments and COVID-19 anxiety, participants’ leaving thresholds for both environments were entered into a regression alongside age, gender and income. The overall model was not significant (*F*_(5,393)_ = 1.14, *p* = .340, R^2^ = .01) and neither the leaving threshold from the poor environment (β= 0.02, t = .034, *p* = .735) nor the rich environment (β = -0.01, t = -0.20, *p* = .84) predicted COVID-19 anxiety.


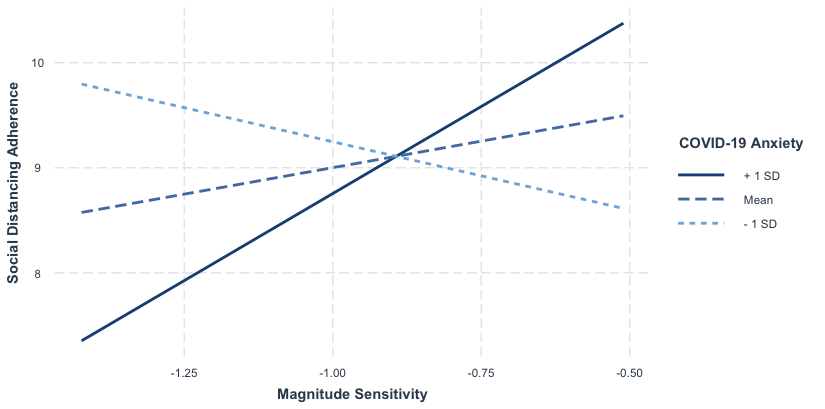


Supplementary Figure 4: Predicted values and fitted lines (± 1 SD) for the interaction between magnitude sensitivity and COVID-19 anxiety on social distancing adherence.

**Exploratory analyses: Cognitive characteristics predict capability, opportunity and motivation to engage in social distancing**

Supplementary Table 8: COM-B scales at established early acute (T1) and established (T2) phases of pandemic

| Measure | T1 score | T2 score | Paired t statistic | df | *p* value |
| --- | --- | --- | --- | --- | --- |
| Motivation | 29.26 (4.20) | 36.54 (6.36) | 25.42 | 440 | < .001 |
| Capability | 12.73 (2.10) | 13.24 (2.15) | 5.59 | 440 | < .001 |
| Opportunity | 23.22 (4.01) | 24.64 (4.38) | 6.69 | 440 | < .001 |
| Total | 64.94 (8.56) | 73.10 (11.14) | 18.23 | 440 | < .001 |

**Supplementary References:**

1. Afifi, A. A., Kotlerman, J. B., Ettner, S. L. & Cowan, M. Methods for Improving Regression Analysis for Skewed Continuous or Counted Responses. *Ann. Rev. Public Health* 28:95-111 (2007).
